# High-throughput sequencing of pituitary and hypothalamic microRNA transcriptome associated with high rate of egg production

**DOI:** 10.1186/s12864-017-3644-3

**Published:** 2017-03-23

**Authors:** Nan Wu, Qing Zhu, Binlong Chen, Jian Gao, Zhongxian Xu, Diyan Li

**Affiliations:** Farm Animal Genetic Resources Exploration and Innovation Key Laboratory of Sichuan Province, Sichuan Agricultural University, Chengdu, People’s Republic of China 610000

**Keywords:** Luhua chicken, Egg-laying, qRT-PCR, SNPs, Reproduction regulation

## Abstract

**Background:**

MicroRNAs exist widely in viruses, plants and animals. As endogenous small non-coding RNAs, miRNAs regulate a variety of biological processes. Tissue miRNA expression studies have discovered numerous functions for miRNAs in various tissues of chicken, but the regulation of miRNAs in chicken pituitary and hypothalamic development related to high and low egg-laying performance has remained unclear.

**Results:**

In this study, using high-throughput sequencing technology, we sequenced two tissues (pituitary and hypothalamus) in 3 high- and 3 low-rate egg production Luhua chickens at the age of 300 days. By comparing low- and high-rate egg production chickens, 46 known miRNAs and 27 novel miRNAs were identified as differentially expressed (*P* < 0.05). Six differentially expressed known miRNAs, which are expressed in both tissues, were used in RT-qPCR validation and SNP detection. Among them, seven SNPs in two miRNA precursors (gga-miR-1684a and gga-miR-1434) were found that might enhance or reduce the production of the mature miRNAs. In addition, 124 and 30 reciprocally expressed miRNA-target pairs were identified by RNA-seq in pituitary and hypothalamic tissues, respectively and randomly selected candidate miRNA and miRNA-target pairs were validated by RT-qPCR in Jiuyuan black fowl. Gene Ontology (GO) and Kyoto Encyclopedia of Genes and Genomes (KEGG) pathway annotation illustrated that a large number of egg laying-related pathways were enriched in the high-rate egg production chickens, including ovarian steroidogenesis and steroid hormone biosynthesis.

**Conclusions:**

These differentially expressed miRNAs and their predicted target genes, especially identified reciprocally expressed miRNA-target pairs, advance the study of miRNA function and egg production associated miRNA identification. The analysis of the miRNA-related SNPs and their effects provided insights into the effects of SNPs on miRNA biogenesis and function. The data generated in this study will further our understanding of miRNA regulation mechanisms in the chicken egg-laying process.

**Electronic supplementary material:**

The online version of this article (doi:10.1186/s12864-017-3644-3) contains supplementary material, which is available to authorized users.

## Background

MicroRNAs (miRNAs) can modulate almost all biological processes at the post-transcriptional level in viruses, plants and animals. miRNAs are a class of small endogenous non-coding RNAs that are approximately 19 to 24 nucleotides in length [1]. miRNAs negatively regulate extensive gene expression through sequence-specific interactions with the 3′untranslated regions (UTRs) of target mRNAs and thereby cause mRNA destabilization or translational repression [2, 3]. Currently, 35,828 mature miRNAs from 223 species have been discovered and deposited in the publicly available miRNA database miRBase (Release 21.0, June 2014) [4]. Genome-wide miRNA expression studies demonstrate that miRNAs have numerous significant biological functions, especially for signaling pathways implicated in development, cellular differentiation, hematopoiesis, proliferation, apoptosis and oncogenesis [1, 5–8].

Chickens (*Gallus gallus domesticus*) are an important model organism that bridges the evolutionary gap between mammals and other vertebrates [9]. Chickens were domesticated in Asia at least by 5400 _BC_, perhaps as early as 8000 _BC_ [10]. Previous studies have reported abundant miRNA identification in various chicken tissues, such as embryo [8, 11–14], ovary and testis [13, 15], lung and trachea [16], somite [17], adipose tissue [18], skeletal muscle [19] and immune organs [20]. Further, some studies have assessed diseases in chicken [21–23]. However, a role for miRNAs in chicken pituitary and hypothalamic development related to high and low egg-laying performance has not been reported clearly. The characteristics of pituitary and hypothalamic tissues are highly related with growth development and reproductive traits of chickens, and they are ideal tissues to identify molecular markers associated with egg production [24].

In the present study, we constructed small-RNA cDNA libraries from pituitary and hypothalamic tissues of Luhua chicken using individuals at the age of 300 days with relatively high and low rates of egg production. In poultry breeding programs, the egg number at 300 days of age is generally used as the most valuable indicator of total egg production potential [25]. Through high-throughput sequencing of small RNA libraries and subsequent bioinformatic analysis, comprehensive miRNA profiles of pituitary and hypothalamic tissues from low and high rate of egg production chickens were generated, and comparative analysis of miRNA data was performed. The discovery of miRNA resources from this study will contribute to a better understanding in miRNAs regulating chicken egg production biological processes. Moreover, we will be able to screen suitable miRNAs for use as molecular markers in the application of genetic selection in the chicken breeding programs.

## Methods

### Experimental animals, tissue collection, rna extraction and sequencing

Eight hundred Luhua chickens from the Experimental Chicken Farm of Sichuan Agricultural University were used in this study. Then, low rate of egg production (LP) chicken and high rate of egg production (HP) chickens were categorized according to their egg number at 250 days of age. The egg production distribution of 800 birds at 300 days of age was shown in Additional file 1: Figure S1. According to the similar reproductive traits and regular egg production cycle, three LP and three HP chickens (Additional file 2: Table S1) were selected for tissue collection at 300 days of age. More detailed animal descriptions, sample grouping and egg production cycle recording methods can be found in our previous study [26].

The pituitary and hypothalamic tissues of six chickens were dissected as described [27], then snap frozen in liquid nitrogen and stored at −80 °C until RNA extraction. The total RNA of pituitary and hypothalamus was isolated by using RNeasy Mini Kit (Qiagen, Hilden, Germany) followed the manufacturer’s instructions. Twelve complementary DNA (cDNA) libraries for small RNA from chicken pituitary and hypothalamic tissues were constructed according to published miRNA cloning protocols [28, 29]. All samples were sequenced by 50-bp paired end reads (PE-50) on an Illumina HiSeq 2500 platform. The Institutional Animal Care and Use Committee of Sichuan Agricultural University approved animal experimentation in this study under permit number DKY- S20143204.

### Sequence analysis

The Illumina 5’ and 3’ sequencing adapters were trimmed with Cutadapt software [30], and the small RNA libraries were further filtered to a minimum length of 17 nt and a maximum length of 35 nt. The clean reads were mapped to the chicken genome (galGal4) using MegaBLAST, and rRNA, tRNA, miscRNA, snRNA and snoRNA were discarded from the small RNA sequences. The remaining sequences were again searched against the miRBase 21.0 database [31–33] of *Gallus gallus* known miRNA sequences with zero or one mismatch. The sequences matching the *Gallus gallus* miRBase database were considered as known miRNA sequences. Next, after filtering known miRNA sequences, the remaining sequences were BLAST searched against the *Gallus gallus* genome. The sequences matching the chicken genome were used to predict the novel miRNA by the mirDeep2 [34–36] using default parameters. These sequences were considered as potential novel miRNAs, and expression of all miRNAs was assayed. Differential expression for known and novel miRNAs were analyzed using edgeR [37]. Reads per million miRNAs mapped (RPM) values were used to represent miRNA expression levels. *P*-values were calculated using right-tailed Fisher’s exact test. *P* < 0.05 and |Log_2_Fold Change| ≥1(LogFC) were used to screen differentially expressed miRNAs. The miRNA target prediction software miRDB [38] was used to predict the binding sites of the differentially expressed miRNA. The TargetScan principle (http://www.targetscan.org/) was also applied in the prediction procedures. The main functions of the predicted target genes regulated by differentially expressed miRNAs were determined using GO and KEGG functional classifications by Blast2GO program [39]. In GO terms, *P*-value ≤ 0.001 was used to identify the significantly enriched GO terms, and the *P*-value cut-off was 0.05 for KEGG terms.

### Validation of miRNA expression by RT-qPCR

To validate the reliability of Illumina analysis, we tested the expression of eight miRNAs expressed in both tissues, including six significantly differentially expressed miRNAs and two non-significant expressed miRNAs, using reverse transcription (RT) Real-time PCR. The RT-qPCR primers were designed using Primer 5.0 (http://downloads.fyxm.net/Primer-Premier-101178.html), and listed in Additional file 2: Table S2 (miRNA-specific primers were synthesized by the Shanghai Biological Technology Co., and universal primers were provided by the miRcute miRNA qPCR Detection kit, Aidlab, Beijing, China). Real-time PCR was performed in a 96-wells plate using a Bio-Rad iQ5 Real-time PCR Detection System (Bio-Rad, California, USA) according to the protocol. In addition, 5.8S rRNA, which has relatively stable expression in most tissues, was used as an endogenous control [40], and the expression level of 5.8S rRNA was used to normalize the RT-qPCR results for each miRNA. All reactions were run in three technical replicates and included negative controls without template. Fold-changes of miRNA expression were calculated using the 2^-ΔΔCt^ method (versus 5.8S rRNA) [41]. All data are expressed as the mean ± standard deviation, and statistical analysis using Student’s *t*-tests was performed with SPSS 16.0 software (SPSS Inc.).

### Validation of candidate miRNA and miRNA-target pairs in the Jiuyuan black fowl

The Jiuyuan black fowl (Gallus Domesticus) is similar with Luhua bird, which has been recognized as a commercial dual-purpose egg-meat type chicken, but Luhua bird has a more superior reproductive performance such as high rates of egg production. Four hundred Jiuyuan black fowls from the Experimental Chicken Farm of Sichuan Agricultural University were used in this study. The processes of samples selection, tissues collection, RNA extraction and RT-qPCR validation of miRNAs and genes expression were according to previous described methods. The reproductive performance information of selected chicken samples was shown in Additional file 2: Table S1 and Additional file 1: Figure S2. The RT-qPCR primers of miRNAs and genes were listed in Additional file 2: Table S2. In addition, the expression levels of 5.8S rRNA and GAPDH were used to normalize the RT-qPCR results for each miRNA and gene, respectively.

### Detection of SNPs in miRNAs

To explore the effects of SNPs on six known miRNAs differentially expressed in both tissues, SNPs in 120 low and 120 high rate of egg production chickens were scanned. Blood samples (0.5 to 1 ml) were collected in a 1-ml syringe primed with EDTA anticoagulation agent. Genomic DNA was phenol-extracted following standard procedures [42]. Six pairs of primer (Additional file 2: Table S2) were used for PCR, and products were sequenced by an ABI 3730xl automatic sequencer. The obtained sequences were aligned with the miRNA precursors using the program Seqman 5.01 of DNAstar Software (DNAstar Inc. Madison, WI, USA) [43]. To study the effect of SNPs on miRNA biogenesis, we calculated the second structure energy of different SNP-type precursors using RNAfold (http://nhjy.hzau.edu.cn/kech/swxxx/jakj/dianzi/Bioinf4/miRNA/miRNA1.htm) [44] and compared the energy changes between SNP-type pre-miRNAs and wild type pre-miRNAs.

## Results

### Overview of miRNA sequencing

In total, 70.23 and 74.02 million raw reads were obtained from LP and HP chickens, respectively. After filtering the low-quality sequences, a total of 64.39 (clean ratio: 91.64%) and 67.20 (clean ratio: 90.85%) million clean reads in LP and HP chickens, respectively, were used for further analysis (Additional file 2: Table S3). Among the clean reads, 50.18 M reads from LP and 51.05 M reads from HP were successfully mapped and annotated, amounting to 77.92% and 75.96% of the total reads, respectively. In addition, 48.13 M reads in LP and 47.88 M reads in HP were found to be similar to miRNAs. HP chickens have more small RNAs and unique small RNAs than LP chicken in both tissues. The remaining of the mapped reads were other types of RNA, including rRNA, tRNA, miscRNA (repeat and polII-transcribed), snRNA and snoRNA (Additional file 2: Table S4). In total small RNAs, most small RNA were miRNA (approximately 70%) followed by unannotated RNAs (approximately 20%). However, in the unique small RNAs, unannotated RNAs account for the biggest proportion (approximately 40%), and miRNA (approximately 30%) ranked second, except in the hypothalamus of HP chickens (Additional file 1: Figure S3). Proportions of the remaining categories of small RNAs, including tRNA, rRNA, miscRNA, snRNA or snoRNA, were relatively lower (less than 2%). The size distribution of small RNAs was similar in all tissues, and the majority of them changed from 20 to 24 nt, which was consistent with the typical size range of small RNAs (Additional file 1: Figure S4). The most abundant size class was 22 nt, which accounted for approximately 10% in the unique reads and 40% in the total reads in the four libraries.

### Expression patterns of known and novel miRNAs in chicken pituitary and hypothalamus

**Fig. 1:**
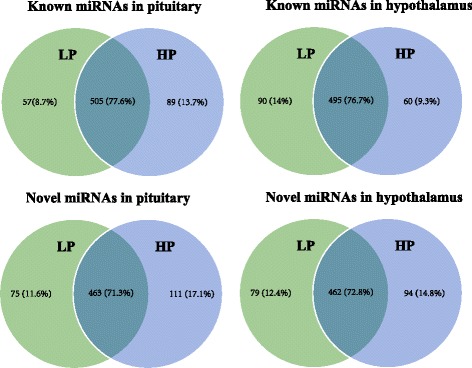
The distribution of known and novel miRNAs in pituitary and hypothalamus tissues

In the present study, a total of 651 (LP:562, HP:594) and 645 known miRNAs (LP:585, HP:555) was identified in pituitary and hypothalamic tissues, respectively (Additional file 2: Table S5). In the pituitary, 505 (77.6%) unique miRNAs were co-expressed in LP and HP chickens; 57 (8.7%) and 89 (13.7%) were specifically expressed in LP and HP chickens, respectively. In the hypothalamus, 495 (76.7%) unique miRNAs were co-expressed in LP and HP chickens; 90 (14%) and 60 (9.3%) were specifically expressed in LP and HP chickens, respectively (Fig. 1). The minimal differences in the specifically expressed miRNAs of LP and HP chickens reflect the organizational complementarity of the two tissues.

A total of 649 novel miRNAs (LP:538, HP:574) and 635 novel miRNAs (LP:541, HP:556) were predicted in pituitary and hypothalamic tissues, respectively (Additional file 2: Table S6). In pituitary, 463 (71.3%) unique miRNAs were co-expressed in LP and HP chickens; 75 (11.6%) and 111 (17.1%) were specifically expressed in LP and HP chickens, respectively. In the hypothalamus, 462 (72.8%) unique miRNAs were co-expressed in LP and HP chickens; 79 (12.4%) and 94 (14.8%) were specifically expressed in LP and HP chickens, respectively (Fig. 1).

In the known miRNA expression profile of the pituitary and hypothalamus, the read numbers of the top 10 miRNAs accounted for 73.65% and 79.26% of the total reads, respectively. In the novel miRNA expression profile, the read numbers of the top 10 miRNAs accounted for 97.35% and 97.63% of the total reads for the pituitary and hypothalamus, respectively. The expression profile revealed that most miRNAs were expressed by a small portion of miRNA genes. Interestingly, although there were some changes in the rank of miRNA expression, the known and novel miRNAs with the highest expression levels were consistent in the two tissues. In known miRNAs, gga-miR-26a-5p displayed greater than 12 M reads and exhibited the highest expression level followed by gga-miR-99a-5p, which displayed greater than 5 M reads. Some miRNAs (such as gga-miR-6516-5p and gga-miR-130a-5p) displayed less than approximately 1,000 reads, indicating that expression significantly varied among different miRNA families, which is consistent with previous studies [45]. In novel miRNAs, we found that the most abundant novel miRNA, gga-novel-1-mature, displayed greater than approximately 0.96 M reads in pituitary, it only displayed approximately 0.39 M reads in hypothalamus. The significant difference of miRNA expression may reflect the miRNA different functions in the two tissues.

Moreover, compared with known miRNAs, the sequencing frequencies of novel miRNAs were considerably reduced. The same expression pattern has also been reported in other species [46, 47], which suggests that novel miRNAs are typically weakly expressed, whereas known miRNA genes are highly expressed.

### miRNA:miRNA* pairs in chicken pituitary and hypothalamus

The miRNA:miRNA* pairs are the mature miRNAs and miRNA*s (miR-#-5p and miR-#-3p) align to the 5′ and 3′ end regions of the precursors, respectively. In the study, a total of 181 duplex like miRNA:miRNA* pairs were obtained from known miRNA sequences of the two tissues (Additional file 2: Table S7). Commonly, the miR-#-5 ps were detected at the same or relatively high expression levels than miR-#-3 ps (Table 1), suggesting that the expression level of miR-#-3p mainly relied on the degradation degree and rate because both strands of miRNA duplex were produced in equal amounts by transcription. However, some miR-#-3p exhibited relatively increased expression levels compared with miR-#-5p (such as gga-miR-92-3p, gga-miR-7471-3p, gga-miR-130c-3p). Recently, some miRNA* sequences (miR-#-3p) with abundant expression were reported as mature functional miRNAs [48]. The relatively high number of reads of these miRNA*s indicates that they may play a functional role in regulating gene expression, such as reproduction regulation, in present study. Such a phenomenon has also been described in several previous studies [46, 47].

**Table 1:** The detail distribution of miRNA:miRNA* pairs in pituitary and hypothalamus tissues

Tissues	Number of miRNA:miRNA*(pairs)	miR-#-5p ≥ miR-#-3p(pairs)	miR-#-5p < miR-#-3p(pairs)	Common in the two tissues(pairs)	Unique in each tissue(pairs)	Total(pairs)
pituitary	163	86	77	145	18	181
hypothalamus	163	87	76		18	

### Hierarchical cluster analysis of differentially expressed miRNAs

**Fig. 2:**
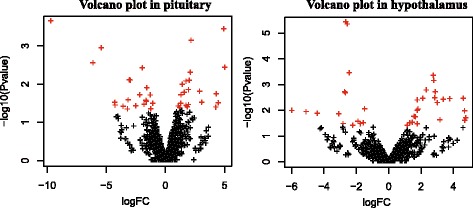
Scatter plot of the high-throughput sequencing data. The high-throughput sequencing data (differentially expressed miRNAs) are graphed on the scatter plot to visualize variations in miRNA expression between HP and LP chickens. Diagrams reflect fold change value (HP/LP) distribution in the differentially expressed miRNA numbers. In MA and volcano plots, red dots represent the differentially expressed miRNAs, whereas black represent miRNAs with similar expression

Correlation analysis revealed high reproducibility in the same group, with Pearson correlation coefficients >0.75 (Additional file 1: Figure S5). By comparing the abundance of known and novel miRNAs between HP and LP chickens (Fig. 2), a total of 46 known miRNAs and 27 novel miRNAs that are differentially expressed (*P* < 0.05) in the two tissues were identified (Additional file 2: Table S8). Specifically, 21 up-regulated and 21 down-regulated miRNAs were identified in pituitary tissues, and 24 up-regulated and 15 down-regulated miRNAs were identified in hypothalamus tissues. In addition, for the absolute values of logFC, the majority of differentially expressed miRNAs exhibit a 1- to 4-fold difference, and 18 miRNAs showed differences greater than 4-fold between the LP and HP in the two tissues. Among the up-regulated miRNAs, gga-novel-148-mature had the highest logFC at 5.01-fold. Among the down-regulated miRNAs, gga-novel-16-mature and gga-novel-220-mature had the highest |logFC| with 9.70-fold, followed by gga-novel-306-mature, gga-miR-1682, gga-miR-1683 and gga-miR-6549-3p, |logFC| with more than 5-fold.

**Fig. 3:**
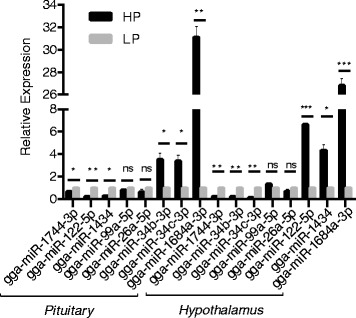
Validation of the miRNA expression profile by qRT-PCR. The relative expression levels of eight selected miRNAs were calculated according to the 2^-ΔΔCt^ method using 5.8S rRNA as an internal reference RNA. Error bars represent the standard deviation. The *x*-axis indicates different miRNAs in the two tissues. *P < 0.05, **P <0.01, ***P < 0.001

To validate the Illumina small RNA deep sequencing data, RT-qPCR detection assays were used to confirm the expression of eight miRNAs expressed in both tissues, including six differentially expressed known miRNAs and two non-significant expressed miRNAs. As shown in Table 2 and Fig. 3, the general expression patterns of eight miRNAs from the Illumina sequencing are consistent with the RT-qPCR results, which further support the reliability of the Illumina sequencing data. The discrepancies with respect to ratio may be attributed to the essentially different algorithms and sensitivities between the two techniques.

**Table 2:** Evaluation of the expression profile variation between RNA-Seq and RT-qPCR for the selected miRNAs

Tissue	miRNA	Fold change (HP/LP)		*P*-value	
		RNA-Seq	RT-qPCR	RNA-Seq	RT-qPCR
pituitary	gga-miR-1744-3p	0.32	0.66	0.0296	0.0279
	gga-miR-122-5p	0.26	0.23	0.0039	0.0095
	gga-miR-1434	0.42	0.28	0.0464	0.0229
	gga-miR-99a-5p	0.87	0.78	0.6680	0.1060
	gga-miR-26a-5p	0.84	0.63	0.5370	0.2780
	gga-miR-34b-3p	3.34	3.53	0.0377	0.0477
	gga-miR-34c-3p	3.86	3.39	0.0362	0.0401
	gga-miR-1684a-3p	30.57	31.12	0.0004	0.0010
hypothalamus	gga-miR-1744-3p	0.15	0.23	0.0021	0.0064
	gga-miR-34b-3p	0.16	0.22	0.0023	0.0012
	gga-miR-34c-3p	0.18	0.14	0.0004	0.0062
	gga-miR-99a-5p	1.34	1.31	0.3620	0.0685
	gga-miR-26a-5p	1.19	0.69	0.5430	0.2510
	gga-miR-122-5p	7.41	6.63	0.0021	0.0005
	gga-miR-1434	4.99	4.35	0.0018	0.0211
	gga-miR-1684a-3p	24.47	26.81	0.0037	0.0006

### Target prediction and Gene Functional Annotation

In total, we predicted 2541 target genes in pituitary and 2108 target genes in hypothalamic tissues (Additional file 2: Table S9). Some predicted targets were likely to be targeted by multiple miRNAs at multiple targeting sites. Typically, tyrosine-protein kinase receptor (*CTK-1*) can be targeted by three miRNAs, including gga-miR-15c-5p, gga-miR-15a and gga-miR-16-5p.

**Fig. 4:**
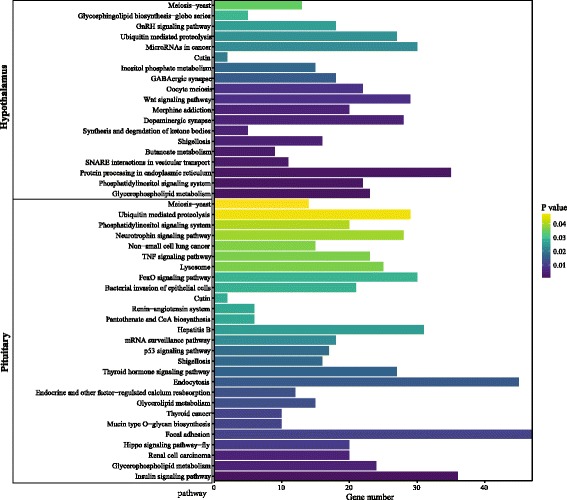
Selected significant pathway annotation in pituitary and hypothalamus tissues

To probe the biological roles of differentially expressed miRNAs, all of the predicted targets of them were mapped to terms in the GO and KEGG databases. In total, 369 GO terms and 28 KEGG pathways in pituitary tissues and 201 GO terms and 19 KEGG pathways in hypothalamic tissues were significantly enriched (Additional file 2: Table S10). GO enrichment analysis revealed that target genes were main functionally enriched in cellular process, cell, cell part and protein binding in the two tissues (Additional file 1: Figure S6). KEGG analysis results showed that most targets in pituitary tissues were mainly involved in focal adhesion, endocytosis, insulin signaling pathway, hepatitis B and FoxO signaling pathway. In hypothalamic tissues, most targets were mainly involved in protein processing in endoplasmic reticulum, microRNAs in cancer, wnt signaling pathway, dopaminergic synapse and ubiquitin mediated proteolysis (Fig. 4). Notably, a specific enrichment of genes was observed in some reproduction regulation pathways, such as ovarian steroidogenesis, oocyte meiosis, GnRH signaling pathways, progesterone-mediated oocyte maturation, calcium signaling pathways, endocrine and other factor-regulated calcium reabsorption, dopaminergic synapse, oxytocin signaling pathway and MAPK signaling pathway (Additional file 2: Table S11).

### The identification of reciprocally expressed miRNA-target pairs

**Fig. 5:**
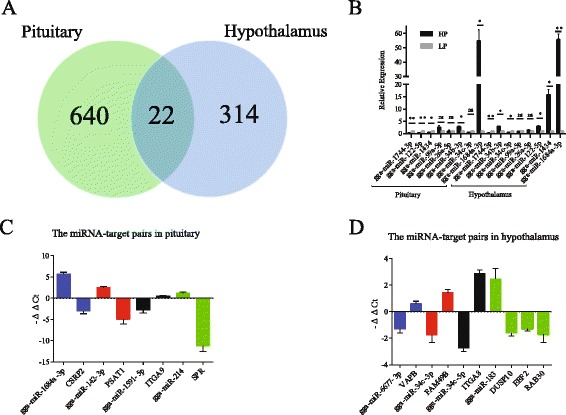
**a** The distribution of differentially expressed genes in pituitary and hypothalamic tissues between low- and high-rate egg production chickens. **b** Validation of eight miRNA expression profile by qRT-PCR in pituitary and hypothalamic tissues of Jiuyuan black fowl. **c**, **d** The expression of randomly selected four miRNA-target pairs in the pituitary and hypothalamic tissues of Jiuyuan black fowl. The same color indicated the miRNA and its corresponding reciprocally expressed target genes. The expression of each miRNA was normalized to 5.8S rRNA and then transformed to a log 2 scale. The expression of each target gene was relative to GAPDH and also transformed to a log 2 scale. All four miRNA-target pairs showed significantly reciprocal expression patterns

In order to further explore the relationship between differentially expressed miRNAs and their predicted targets, we added a transcriptome analysis in pituitary and hypothalamic tissues of the six samples using RNA-seq. A total of 662 and 336 differentially expressed genes (*P* < 0.05) were identified in pituitary and hypothalamic tissues, respectively (Fig. 5a and Additional file 2: Table S12). By comparing miRNAs predicted targets and these differentially expressed genes, 124 and 30 miRNA-target pairs demonstrated a reciprocal expression pattern in pituitary and hypothalamic tissues, respectively (Additional file 2: Table S13). KEGG analysis were also conducted on these miRNA-target pairs, and most mapped pathways were demonstrated to play important roles in regulating metabolism, development, reproduction, tumorigenesis and many other processes. These predicted reciprocally expressed miRNA-target pairs will provide invaluable insights into candidate miRNAs and genes for reproductive traits and selective breeding of chicken.

### Influence of SNPs in miRNAs on the energy of the miRNA secondary structure

Mutations in miRNAs or in their target sites have been demonstrated to potentially enhance or interrupt miRNA biogenesis or target alteration [49–52], resulting in phenotypic changes associated with diseases or traits [51, 53, 54]. To date, the effects of SNPs on miRNA biogenesis and regulation in chickens have not been reported. In the present study, using conventional Sanger sequencing, we PCR-amplified and sequenced six precursors of known miRNAs, gga-miR-1684a-3p, gga-miR-1744-3p, gga-miR-34b-3p, gga-miR-34c-3p, gga-miR-122-5p and gga-miR-1434, which are differentially expressed in both tissues. All the precursor regions were successfully covered. When the Sanger reads were compared with the reference precursors, we found that two miRNA precursors, gga-mir-1684a and gga-mir-1434, were identified as containing SNPs. In total, 83 haplotypes, 81 SNP sites and 112 SNPs were successfully detected (Table 3). By comparison of statistical significance, we found that the distribution of haplotypes showed no significant differences between HP and LP chickens. However, regarding the distribution of SNP sites and SNP numbers, HP chickens had significantly greater SNP sites and SNP numbers compared with LP chickens. These data indicate that HP chickens with a high egg-laying trait have more mutations in the miRNA biogenesis, which may have special physiological significance in the egg-laying process.

**Table 3:** Overview of PCR data processing of all sequencing samples

miRNA	Precursor/Stem-loop sequence	Gene family	Accession	Number of samples	Number of haplotypes	*P*-value	Number of polymorphic sites	*P*-value	Number of SNP	*P*-value
gga-miR-1684a-3p	gga-mir-1684a	mir-1684	MIMAT0007572	HP:71 LP:73	HP:16 LP:19 Total:28	0.6252	HP:34 LP:15 Total:35	0.0005***	HP:40 LP:17 Total:44	5.0214E-05***
gga-miR-1434	gga-mir-1434	mir-1434	MIMAT0007295	HP:100 LP:111	HP:25 LP:34 Total:55	0.3629	HP:45 LP:31 Total:46	0.0099**	HP:61 LP:38 Total:68	0.0001***

*P < 0.05, **P <0.01, ***P < 0.001 meant significant difference between the HP and LP chickens

Next, we investigated the effect of the SNPs on the energy change (ΔΔG) of the secondary structures of the two precursors. Given the large sample sizes for SNP identification and to avoid the calculation of too many SNPs and effect of sequence error, haplotypes in which the sample size was less than 3 were not considered. In total, we statistically analyzed 10 haplotypes, 8 SNP sites and 10 SNPs in the two miRNA precursors (Additional file 2: Table S14). We found that an SNP in the mature regions did not change the energy of the structure, whereas the remaining 9 SNPs changed the energy of the predicted secondary structures (Table 4). For seven of the SNPs, the absolute energy change values were ≥ 0.3 kcal/mol, which is the minimum energy change reported to be required to change the production of mature miRNAs [49]. Gong et al. summarized the rule that if an SNP increases the hairpin structure energy, the production of the mature miRNA will increase. If the SNP decreases the energy, the production of the mature miRNA will be reduced [55]. The identified seven SNPs therefore enhance or reduce the production of the mature miRNAs and may play special physiological roles in the egg-laying process.

**Table 4:** Effect of SNPs on miRNA precursor energy changes

Precursor	SNP position in Precursor	SNP	SNP location	ΔΔG (kcal/mol)
gga-mir-1684a	6	C→G	stem	−1.9
	20	G→U	stem	5.0
	38	C→U	anti-stem	1.3
	39	A→G	anti-stem	−0.2
	43	U→C	loop	−2.7
	78	A→G	mature	−2.8
	78	A→C	mature	−6.9
gga-miR-1434	19	A→G	mature	0
	71	G→A	anti-stem	−0.1
	71	G→C	anti-stem	4.3

*MFE* minimum free energy

*ΔΔGS* the MFE difference value between wild-type precursors and SNP-type precursors. The minus value indicated that the SNP-type precursors had lower structure energy than the wild precursors. Otherwise, the former had higher structure energy than the latter

### Validation of candidate miRNAs and miRNA-target pairs expression in the Jiuyuan black fowl

In the present study, in order to explore the applicability of these identified candidate miRNAs on other native birds, we detected the expression of previously selected eight candidate miRNAs in pituitary and hypothalamic tissues of Jiuyuan black fowl. Although the ratio between LP and HP chickens for each miRNA may be different, the expression pattern of most selected miRNAs in Jiuyuan black fowl is consistent with the high-throughput sequencing results of Luhua chicken except gga-miR-34c-3p shown no difference in pituitary and gga-miR-34b-3p shown up-regulated pattern in hypothalamus (Fig. 5b).

In addition, we also validated the expression of four egg production-related miRNA-target pairs which were randomly selected from identified miRNA-target pairs of Luhua chickens using RT-qPCR in pituitary and hypothalamic tissues of Jiuyuan black fowl. All four pairs showed significantly reciprocal expression patterns (Fig. 5c, d), consistent with the observation that miRNAs predominantly function to decrease target gene levels [56–58].

## Discussion

The miRNA sequences we identified were generally close to 22 nucleotides in size, which is consistent with previous reports [59, 60], and the annotation of RNA distribution revealed that the clean reads were highly enriched and included a myriad of miRNA sequences. Correlation analysis revealed that the twelve samples have high repeatability in the same group and were obviously different in different group. In addition, abundant known and novel miRNAs are identified and predicted in the pituitary and hypothalamic tissues. By comparing HP and LP chickens, 46 known miRNAs and 27 novel miRNAs that are differentially expressed (*P* < 0.05) in the two tissues were found. Moreover, the RT-qPCR results provide evidence that Illumina small RNA deep sequencing is a sensitive and reliable approach to identifying differentially expressed miRNAs in chicken pituitary and hypothalamic tissues. In summary, all of the above results suggested that the deep sequence data are representative and reliable for the subsequent analyses.

In pituitary and hypothalamic libraries, gga-miR-26a-5p (>12 M reads) and gga-miR-99a-5p (>5 M reads) were the most two frequently sequenced miRNAs. Despite the different read numbers in the two miRNAs, this result is consisted with our previous study [26]. miR-26a regulates tissue and cell growth and differentiation [61, 62] and has anti-apoptotic effects on many cancers [63–65]. mir-99a and mir-99b inhibit proliferation of c-Src-transformed cells and prostate cancer cells by targeting mTOR [66, 67] and were identified as novel targets in some important biological process, such as the TGF-β-induced epithelial to mesenchymal transition [68]. Among other miRNAs, we found that the let-7 miRNA family was another abundant cluster with let-7f-5p being the most abundantly expressed miRNA. The let-7 miRNA family is abundantly expressed in bovines [69–71] and in murine ovaries and testis [72]. Furthermore, gga-miR-7, gga-miR-148a-3p, gga-miR-146c-5p, gga-miR-125b-5p, gga-miR-30d, gga-miR-153-3p and gga-miR-126-3p were abundant in our sequencing libraries and have been shown to occur in other animals [15, 69, 73, 74]. The significant biological functions of these miRNAs imply that they have important roles in the female reproductive physiology of chicken.

In significantly differentially expressed miRNAs, some miRNAs play important roles in various aspects. For example, miR-138 [75–77], miR-29a [78–81], miR-490-3p [82, 83], miR-9-3p [84–86], and miR-135 [87–90] have pivotal roles in tumorigenesis and tumor progression by acting as tumor suppressors. miR-138 modulates the DNA damage response by repressing histone H2AX expression [91]. miR-490-3p [92] and miR-135 [93] as potential regulators of myogenesis also modulate the proliferation of muscle cells and are involved in skeletal muscle development. The important roles of these miRNAs and their significant expression pattern between LP and HP suggested that they might have important physiological roles in the process of chicken reproduction.

Furthermore, we found eight significantly differentially expressed miRNAs in both tissues, including six known miRNAs (gga-miR-1684a-3p, gga-miR-1744-3p, gga-miR-34b-3p, gga-miR-34c-3p, gga-miR-122-5p and gga-miR-1434) and two novel miRNAs (gga-novel-367-mature and gga-novel-465-mature). Intriguingly, among the eight miRNAs, gga-miR-1684a-3p and gga-miR-1744-3p have the same expression pattern, and the remaining six miRNAs exhibited a contrasting expression pattern between the two tissues. We speculated the contrary expression pattern of these candidate miRNAs may be beneficial to the improvement of egg-laying in hens. In addition, miRNA target identification is important to explore the functions of the miRNAs. In the present study, in order to further explore the relationship between differentially expressed miRNAs and their predicted targets, we added a transcriptome analysis of pituitary and hypothalamic tissues to identify reciprocally expressed miRNA-target pairs. These successfully identified miRNA-target pairs suggested they have highly possibility to be related in the egg-laying process. This will provide a great convenience for future studies to functionally validate these miRNA targets. These miRNA-target pairs will also provide invaluable insights into candidate miRNAs and genes for reproductive traits and selective breeding of chicken.

The miR-34 family was significantly differentially expressed between LP and HP chickens in the two tissues. The results were also presented in our previous study [26]. Compared with LP chickens, gga-miR-34b-3p and gga-miR-34c-3p exhibited a significant increase in HP pituitary tissues. In HP hypothalamus, gga-miR-34b (include 3p and 5p) and gga-miR-34c (include 3p and 5p) exhibited significant reductions. Previous studies reported that the mir-34 family was identified as a p53 target and a potential tumor suppressor to regulate processes, such as proliferation, cell cycle, apoptosis and metastasis [94–100]. Some studies also observed that miR-34 can regulate age-associated events and long-term brain integrity to modulate aging and neurodegeneration in *Drosophila* [101]. In the present study, gga-miR-34 were significantly differentially expressed between LP and HP chickens, suggesting that gga-miR-34 has an important impact on reproductive process of hens. However, the contrary expression pattern between pituitary and hypothalamus tissues indicates that the different members of miR-34 family play different roles in the two tissues in the egg-laying process.

In significantly enriched KEGG pathways, the most overrepresented pathways belonged to the metabolic pathways, such as glycerophospholipid metabolism, glycerolipid metabolism and butanoate metabolism. Some pathways associated with endocytosis, cancer, oocyte meiosis, focal adhesion, protein processing in endoplasmic reticulum, cutin, snare interactions in vesicular transport endocrine, other factor-regulated calcium reabsorption, dopaminergic synapse, GABAergic synapse and some signal transduction pathways, such as Renin-angiotensin system and GnRH signaling pathway, were all significantly enriched, indicating the role of the differentially expressed miRNAs in the regulation of cell motility, cell proliferation, the cytoskeleton, cell nutrition, nervous system development and function, communication between cells and the extracellular matrix. Moreover, the insulin signaling pathway was also enriched in our results. Insulin is the most potent anabolic hormone, mediating a wide spectrum of biological responses, including the synthesis and storage of carbohydrates, proteins and lipids and inhibiting their degradation and release back into circulation [102]. In addition, a small number of pathways involved in shigellosis, bacterial invasion of epithelial cells and hepatitis B suggested that the hens were involved in a stage of immune regulation. In general, the results indicated that these differentially expressed miRNAs were mainly involved in cell proliferation and development, signal transduction, metabolic and immune processes and nervous system development and function.

In these KEGG pathways, a specific enrichment of predicted targeted genes was involved in some reproduction related pathways, such as ovarian steroidogenesis, oocyte meiosis and GnRH signaling pathways. Some of these genes play important roles in ovary development and the reproductive management of hens, such as *FSHβ. FSHβ* produces the pituitary glycoprotein hormone FSH, which plays a key role in the reproductive system of chickens, including steroidogenesis, folliculogenesis and follicular maturation [103]. In addition, the significance level of some reproduction regulation-related pathways in the hypothalamus were increased compared with pituitary tissues, such as GnRH signaling pathway and dopaminergic synapse, suggesting that the hypothalamic tissues play a greater role in reproduction regulation-related activities compared with pituitary tissues in hens. Moreover, we found that most identified miRNA-target pairs participated in reproduction regulation-related pathways, suggesting that these miRNA-target pairs were closely associated with high egg-laying performance in chickens. Also, these candidate miRNA and miRNA-target pairs were validated in Jiuyuan black fowl suggested the highly applicability of these egg production-related miRNA-target pairs in other chicken breeds. These identified miRNA-target pairs will provide an opportunity for early high egg-laying performance determination and to select individuals with rapid growth and disease resistance for breeding purposes. Thus, it is important for future studies to functionally validate these miRNA-target pairs. In addition, most of these miRNAs have not been reported their biological function. This information provides a guideline to explore their unknown roles in the reproductive management of hens.

## Conclusions

The current study demonstrated that a diverse and dynamic set of miRNA is expressed in the pituitary and hypothalamic tissues during the egg-laying process in hens. By comparing miRNA expression between low and high rate of egg production chicken in each tissue, miRNAs with significant differences were screened as key factors to participate in the pituitary and hypothalamic reproduction regulatory mechanisms. A comprehensive analysis of the pituitary and hypothalamic microRNA transcriptomes was performed. Plenty of reciprocally expressed miRNA-target pairs were identified and randomly selected candidate miRNA and miRNA-target pairs were validated by RT-qPCR in Jiuyuan black fowl. This information will aid greatly in understanding the complexity of miRNA regulation at egg-laying stages of hens. Furthermore, previous reports demonstrated that chicken miRNA share a high degree of homology with other vertebrate species; therefore, knowledge of miRNA in chicken tissues will also assist in vertebrate development studies and enhance the understanding of the functions of miRNA and regulatory mechanisms of miRNA expression during vertebrate development.

## Additional files

Additional file 1: Figure S1. The egg production distribution of 800 Luhua birds at 300 days of age. **Figure S2.** The egg production distribution of 400 Jiuyuan black fowls at 300 days of age. **Figure S3.** Pie charts of small RNA percentages. **Figure S4.** Length distribution of small RNA in pituitary and hypothalamic libraries of LP and HP chickens. **Figure S5.** Correlation analysis of miRNA expression across samples of LP and HP chickens. **Figure S6.** Gene ontology statistics. The GO classification map includes the top 30 significantly enriched GO terms, which can be used to highlight significant differences in gene function. (PDF 1072 kb)

Additional file 2: Table S1. The main reproductive traits of the selected LP and HP samples. **Table S2.** Primer sequences for real-time PCR validation and SNP detection. **Table S3.** Overview of miRNA-seq data of all samples. **Table S4.** Identities of various RNA sequences in pituitary and hypothalamic tissues. **Table S5.** Expression profile of known miRNAs in pituitary and hypothalamic tissues of low- and high-rate egg production chickens. **Table S6.** Novel miRNAs expressed in pituitary and hypothalamus tissues of low- and high-rate egg production chickens. **Table S7.** The distribution of miRNA pairs in pituitary and hypothalamic tissues. **Table S8.** Differentially expressed miRNAs between low- and high-rate egg production chickens in pituitary and hypothalamic tissues. **Table S9.** The prediction of the differentially expressed miRNA targets in pituitary and hypothalamic tissues. **Table S10.** GO and KEGG pathway annotations for the miRNA targets in pituitary and hypothalamic tissues. **Table S11.** miRNAs and their predicted target genes involved in the regulation processes of reproduction. **Table S12.** Differentially expressed genes between low- and high-rate egg production chickens in pituitary and hypothalamic tissues. **Table S13.** The predicted miRNA-target pairs that are differentially and reciprocally expressed in pituitary and hypothalamic tissues. **Table S14.** The haplotype distribution and sequence variations in the selected miRNA precursors. (XLSX 1731 kb)

## Abbreviations

cDNA: Complementary DNA
GO: Gene Ontology
HP: High rate of egg production chicken
KEGG: Kyoto Encyclopedia of Genes and Genomes
LogFC: Log2Fold Change
LP: Low rate of egg production chicken
MFE: Minimum free energy
miRNA: MicroRNA
PE-50: 50-bp paired end reads
RPM: Reads per million miRNAs mapped
RT-qPCR: Reverse transcription Real-time PCR
SNP: Single Nucleotide Polymorphisms
UTR: Untranslated region
ΔΔG: the MFE difference value between wild-type precursors and SNP-type precursors

## References

[1] Bartel DP (2004). MicroRNAs: genomics, biogenesis, mechanism, and function. Cell.

[2] Bushati N, Cohen SM (2007). microRNA functions. Annu Rev Cell Dev Biol.

[3] Allen E, Xie Z, Gustafson AM, Carrington JC (2005). microRNA-directed phasing during trans-acting siRNA biogenesis in plants. Cell.

[4] Kozomara A, Griffiths-Jones S (2014). miRBase: annotating high confidence microRNAs using deep sequencing data. Nucleic Acids Res.

[5] Mansfield JH, Harfe BD, Nissen R, Obenauer J, Srineel J, Chaudhuri A, Farzan-Kashani R, Zuker M, Pasquinelli AE, Ruvkun G (2004). MicroRNA-responsive ‘sensor’ transgenes uncover Hox-like and other developmentally regulated patterns of vertebrate microRNA expression. Nat Genet.

[6] Lim LP, Glasner ME, Yekta S, Burge CB, Bartel DP (2003). Vertebrate microRNA genes. Science.

[7] Lee SI, Lee BR, Hwang YS, Lee HC, Rengaraj D, Song G, Park TS, Han JY (2011). MicroRNA-mediated posttranscriptional regulation is required for maintaining undifferentiated properties of blastoderm and primordial germ cells in chickens. Proc Natl Acad Sci U S A.

[8] Darnell DK, Kaur S, Stanislaw S, Konieczka JH, Yatskievych TA, Antin PB (2006). MicroRNA expression during chick embryo development. Dev Dyn.

[9] LaDeana WH, Webb M, Ewan B, Wesley W, Ross CH, Chris PP, Peer B, David WB, Martien AM, Mary ED, et al. Sequence and comparative analysis of the chicken genome provide unique perspectives on vertebrate evolution. Nature. 2004;432(7018):695–716. 10.1038/nature03154 15592404

[10] Tixier-Boichard M, Bed'Hom B, Rognon X. Chicken domestication: from archeology to genomics. C R Biol. 2011;334(3):197–204. 10.1016/j.crvi.2010.12.012 21377614

[11] Glazov EA, Cottee PA, Barris WC, Moore RJ, Dalrymple BP, Tizard ML (2008). A microRNA catalog of the developing chicken embryo identified by a deep sequencing approach. Genome Res.

[12] Hicks JA, Tembhurne P, Liu HC (2008). MicroRNA expression in chicken embryos. Poult Sci.

[13] Bannister SC, Tizard ML, Doran TJ, Sinclair AH, Smith CA (2009). Sexually dimorphic microRNA expression during chicken embryonic gonadal development. Biol Reprod.

[14] Burnside J, Ouyang M, Anderson A, Bernberg E, Lu C, Meyers BC, Green PJ, Markis M, Isaacs G, Huang E (2008). Deep sequencing of chicken microRNAs. BMC Genomics.

[15] Kang L, Cui X, Zhang Y, Yang C, Jiang Y (2013). Identification of miRNAs associated with sexual maturity in chicken ovary by Illumina small RNA deep sequencing. BMC Genomics.

[16] Wang Y, Brahmakshatriya V, Zhu H, Lupiani B, Reddy SM, Yoon BJ, Gunaratne PH, Kim JH, Chen R, Wang J (2009). Identification of differentially expressed miRNAs in chicken lung and trachea with avian influenza virus infection by a deep sequencing approach. BMC Genomics.

[17] Rathjen T, Pais H, Sweetman D, Moulton V, Munsterberg A, Dalmay T (2009). High throughput sequencing of microRNAs in chicken somites. FEBS Lett.

[18] Hicks JA, Trakooljul N, Liu HC (2010). Discovery of chicken microRNAs associated with lipogenesis and cell proliferation. Physiol Genomics.

[19] Li T, Wang S, Wu R, Zhou X, Zhu D, Zhang Y (2012). Identification of long non-protein coding RNAs in chicken skeletal muscle using next generation sequencing. Genomics.

[20] Hicks JA, Tembhurne PA, Liu HC (2009). Identification of microRNA in the developing chick immune organs. Immunogenetics.

[21] Tian F, Luo J, Zhang H, Chang S, Song J (2012). MiRNA expression signatures induced by Marek’s disease virus infection in chickens. Genomics.

[22] Wang Q, Gao Y, Ji X, Qi X, Qin L, Gao H, Wang Y, Wang X (2013). Differential expression of microRNAs in avian leukosis virus subgroup J-induced tumors. Vet Microbiol.

[23] Yu Y, Zhang H, Tian F, Bacon L, Zhang Y, Zhang W, Song J (2008). Quantitative evaluation of DNA methylation patterns for ALVE and TVB genes in a neoplastic disease susceptible and resistant chicken model. PLoS One.

[24] Padmanabhan V, Karsch FJ, Lee JS (2002). Hypothalamic, pituitary and gonadal regulation of FSH. Reproduction (Cambridge, England).

[25] Li DY, Zhang L, Smith DG, Xu HL, Liu YP, Zhao XL, Wang Y, Zhu Q (2013). Genetic effects of melatonin receptor genes on chicken reproductive traits. Czeh J Anim Sci.

[26] Wu N, Gaur U, Zhu Q, Chen B, Xu Z, Zhao X, Yang M, Li D (2017). Expressed microRNA associated with high rate of egg production in chicken ovarian follicles. Anim Gen.

[27] Kuo YM, Shiue YL, Chen CF, Tang PC, Lee YP (2005). Proteomic analysis of hypothalamic proteins of high and low egg production strains of chickens. Theriogenology.

[28] Lagos-Quintana M, Rauhut R, Lendeckel W, Tuschl T (2001). Identification of novel genes coding for small expressed RNAs. Science (New York, NY).

[29] Lau NC, Lim LP, Weinstein EG, Bartel DP (2001). An abundant class of tiny RNAs with probable regulatory roles in Caenorhabditis elegans. Science (New York, NY).

[30] Martin M (2011). Cutadapt removes adapter sequences from high-throughput sequencing reads. EMBnet J.

[31] Ambros V, Bartel B, Bartel DP, Burge CB, Carrington JC, Chen X, Dreyfuss G, Eddy SR, Griffiths-Jones S, Marshall M (2003). A uniform system for microRNA annotation. RNA (New York, NY).

[32] Griffiths-Jones S (2004). The microRNA Registry. Nucleic Acids Res.

[33] Griffiths-Jones S, Grocock RJ, van Dongen S, Bateman A, Enright AJ (2006). miRBase: microRNA sequences, targets and gene nomenclature. Nucleic Acids Res.

[34] Friedlander MR, Mackowiak SD, Li N, Chen W, N R (2012). miRDeep2 accurately identifies known and hundreds of novel microRNA genes in seven animal clades. Nucleic Acids Res.

[35] Griffiths-Jones S, Saini HK, van Dongen S, Enright AJ (2008). miRBase: tools for microRNA genomics. Nucleic Acids Res.

[36] Mackowiak SD: Identification of novel and known miRNAs in deep-sequencing data with miRDeep2. Curr Prot Bioinforma, 2011;Chapter 12:Unit 12.10. 10.1002/0471250953.bi1210s36 22161567

[37] Robinson MD, McCarthy DJ, Smyth GK (2010). edgeR: a Bioconductor package for differential expression analysis of digital gene expression data. Bioinformatics.

[38] Wong N, Wang X (2015). miRDB: an online resource for microRNA target prediction and functional annotations. Nucleic Acids Res.

[39] Conesa A, Gotz S, Garcia-Gomez JM, Terol J, Talon M, Robles M (2005). Blast2GO: a universal tool for annotation, visualization and analysis in functional genomics research. Bioinformatics (Oxford, England).

[40] Elela SA, Nazar RN (1997). Role of the 5.8 S rRNA in ribosome translocation. Nucleic Acids Res.

[41] Schmittgen TD, Livak KJ (2008). Analyzing real-time PCR data by the comparative CT method. Nat Protoc.

[42] Saito H, Miura KI. Preparation of transforming deoxyribonucleic acid by phenol treatment. Biochim Biophys Acta. 1963;72(4):619–29. 14071565

[43] Swindell SR, Plasterer TN (1997). SEQMAN. Contig assembly. Methods Mol Biol.

[44] Hofacker IL (2003). Vienna RNA secondary structure server. Nucleic Acids Res.

[45] Gu Y, Zhang L, Chen X (2014). Differential expression analysis of paralichthys olivaceus microRNAs in adult ovary and testis by deep sequencing. Gen Comp Endocrinol.

[46] Xu Z, Chen J, Li X, Ge J, Pan J, Xu X (2013). Identification and characterization of microRNAs in channel catfish (Ictalurus punctatus) by using Solexa sequencing technology. PLoS One.

[47] Chi W, Tong C, Gan X, He S (2011). Characterization and comparative profiling of MiRNA transcriptomes in bighead carp and silver carp. PLoS One.

[48] Guo L, Lu Z (2010). The fate of miRNA* strand through evolutionary analysis: implication for degradation as merely carrier strand or potential regulatory molecule?. PLoS One.

[49] Sun G, Yan J, Noltner K, Feng J, Li H, Sarkis DA, Sommer SS, Rossi JJ (2009). SNPs in human miRNA genes affect biogenesis and function. RNA (New York, NY).

[50] Duan R, Pak C, Jin P (2007). Single nucleotide polymorphism associated with mature miR-125a alters the processing of pri-miRNA. Hum Mol Genet.

[51] Ryan BM, Robles AI, Harris CC (2010). Genetic variation in microRNA networks: the implications for cancer research. Nat Rev Cancer.

[52] Li Q-L, Ju Z-H, Huang J-M, Li J-B, Li R-L, Hou M-H, Wang C-F, Zhong J-F (2011). Two novel SNPs in HSF1 gene are associated with thermal tolerance traits in Chinese Holstein cattle. DNA Cell Biol.

[53] Clop A, Marcq F, Takeda H, Pirottin D, Tordoir X, Bibé B, Bouix J, Caiment F, Elsen J-M, Eychenne F (2006). A mutation creating a potential illegitimate microRNA target site in the myostatin gene affects muscularity in sheep. Nat Genet.

[54] Chen K, Rajewsky N (2006). Natural selection on human microRNA binding sites inferred from SNP data. Nat Genet.

[55] Gong J, Tong Y, Zhang HM, Wang K, Hu T, Shan G, Sun J, Guo AY (2012). Genome-wide identification of SNPs in microRNA genes and the SNP effects on microRNA target binding and biogenesis. Hum Mutat.

[56] Guo H, Ingolia NT, Weissman JS, Bartel DP (2010). Mammalian microRNAs predominantly act to decrease target mRNA levels. Nature.

[57] Doench JG, Sharp PA (2004). Specificity of microRNA target selection in translational repression. Genes Dev.

[58] Brennecke J, Stark A, Russell RB, Cohen SM (2005). Principles of microRNA-target recognition. PLoS Biol.

[59] Yuan L, Zhang X, Li L, Jiang H, Chen J (2014). High-throughput sequencing of microRNA transcriptome and expression assay in the sturgeon, Acipenser schrenckii. PLoS One.

[60] Li R, Zhang CL, Liao XX, Chen D, Wang WQ, Zhu YH, Geng XH, Ji DJ, Mao YJ, Gong YC (2015). Transcriptome microRNA profiling of bovine mammary glands infected with Staphylococcus aureus. Int J Mol Sci.

[61] Wong CF, Tellam RL (2008). MicroRNA-26a targets the histone methyltransferase enhancer of zeste homolog 2 during myogenesis. J Biol Chem.

[62] Luzi E, Marini F, Sala SC, Tognarini I, Galli G, Brandi ML (2008). Osteogenic differentiation of human adipose tissue-derived stem cells is modulated by the miR-26a targeting of the SMAD1 transcription factor &dagger; &dagger. J Bone Min Res Off J Amer Soc Bone Min Res.

[63] Zhang B, Liu XX, He JR, Zhou CX, Guo M, He M, Li MF, Chen GQ, Zhao Q (2011). Pathologically decreased miR-26a antagonizes apoptosis and facilitates carcinogenesis by targeting MTDH and EZH2 in breast cancer. Carcinogenesis.

[64] Garzon R, Fabbri M, Cimmino A, Calin GA, Croce CM (2006). MicroRNA expression and function in cancer. Trends Mol Med.

[65] Saito Y, Jones PA (2006). Epigenetic activation of tumor suppressor microRNAs in human cancer cells. Cell Cycle.

[66] Oneyama C, Ikeda J, Okuzaki D, Suzuki K, Kanou T, Shintani Y, Morii E, Okumura M, Aozasa K, Okada M (2011). MicroRNA-mediated downregulation of mTOR/FGFR3 controls tumor growth induced by Src-related oncogenic pathways. Oncogene.

[67] Sun D, Lee YS, Malhotra A, Kim HK, Matecic M, Evans C, Jensen RV, Moskaluk CA, Dutta A (2011). miR-99 family of MicroRNAs suppresses the expression of prostate-specific antigen and prostate cancer cell proliferation. Cancer Res.

[68] Turcatel G, Rubin N, El-Hashash A, Warburton D (2012). MIR-99a and MIR-99b modulate TGF-β induced epithelial to mesenchymal plasticity in normal murine mammary gland cells. Plos One.

[69] Tripurani SK, Xiao C, Salem M, Yao J (2010). Cloning and analysis of fetal ovary microRNAs in cattle. Anim Reprod Sci.

[70] Huang J, Ju Z, Li Q, Wang C, Hou Q, Li J, Li R, Hou M, Zhong J (2011). Solexa sequencing of novel and differentially expressed microRNAs in testicular and ovarian tissues in Holstein cattle. Int J Biol Sci.

[71] Miles JR, Mcdaneld TG, Wiedmann RT, Cushman RA, Echternkamp SE, Vallet JL, Smith TPL (2012). MicroRNA expression profile in bovine cumulus–oocyte complexes: possible role of let-7 and miR-106a in the development of bovine oocytes ✰. Anim Reprod Sci.

[72] Reid JG, Nagaraja AK, Lynn FC, Drabek RB, Muzny DM, Shaw CA, Weiss MK, Naghavi AO, Khan M, Zhu H (2008). Mouse let-7 miRNA populations exhibit RNA editing that is constrained in the 5′-seed/cleavage/anchor regions and stabilize predicted mmu-let-7a:mRNA duplexes. Genome Res.

[73] Takuya M, Takami T, Shan-Shun L, Osamu I, Yutaka K, Yoshiaki M, Tomoko I, Miki M, Tomohiro K, Tadashi G (2008). MicroRNA (miRNA) cloning analysis reveals sex differences in miRNA expression profiles between adult mouse testis and ovary. Reproduction (Cambridge, England).

[74] Hossain MM, Ghanem N, Hoelker M, Rings F, Phatsara C, Tholen E, Schellander K, Tesfaye D (2009). Identification and characterization of miRNAs expressed in the bovine ovary. BMC Genomics.

[75] Mitomo S, Maesawa C, Ogasawara S, Iwaya T, Shibazaki M, Yashima-Abo A, Kotani K, Oikawa H, Sakurai E, Izutsu N (2008). Downregulation of miR-138 is associated with overexpression of human telomerase reverse transcriptase protein in human anaplastic thyroid carcinoma cell lines. Cancer Sci.

[76] Lu J, Yang D, Liu X, Cheng W, Wang A, Chen Z, Heidbreder CE, Kolokythas A, Zhou X (2011). Identification and experimental validation of G protein alpha inhibiting activity polypeptide 2 (GNAI2) as a microRNA-138 target in tongue squamous cell carcinoma. Hum Genet.

[77] Zhang H, Zhang H, Zhao M, Lv Z, Zhang X, Qin X, Wang H, Wang S, Su J, Lv X (2013). MiR-138 inhibits tumor growth through repression of EZH2 in non-small cell lung cancer. Cell Phys Biochem Int J Exp Cell Phys Biochem Pharmacol.

[78] Tréhoux S, Lahdaoui F, Delpu Y, Renaud F, Leteurtre E, Torrisani J, Jonckheere N, Seuningen IV (2015). Micro-RNAs miR-29a and miR-330-5p function as tumor suppressors by targeting the MUC1 mucin in pancreatic cancer cells. Biochim Biophys Acta.

[79] Desjobert C, Renalier MH, Bergalet J, Dejean E, Joseph N, Kruczynski A, Soulier J, Espinos E, Meggetto F, Cavaillé J (2011). MiR-29a down-regulation in ALK-positive anaplastic large cell lymphomas contributes to apoptosis blockade through MCL-1 overexpression. Blood.

[80] Al-Ahmadi W, Al-Ghamdi M, Al-Souhibani N, Khabar KS (2013). miR-29a inhibition normalizes HuR over-expression and aberrant AU-rich mRNA stability in invasive cancer. J Pathol.

[81] Cui Y, Su WY, Xing J, Wang YC, Wang P, Chen XY, Shen ZY, Cao H, Lu YY, Fang JY (2011). MiR-29a inhibits cell proliferation and induces cell cycle arrest through the downregulation of p42.3 in human gastric cancer. Plos One.

[82] Jia Z, Liu Y, Gao Q, Han Y, Zhang G, Xu S, Cheng K, Zou W (2016). miR-490-3p inhibits the growth and invasiveness in triple-negative breast cancer by repressing the expression of TNKS2. Gene.

[83] Shen J, Xiao Z, Wu WK, Wang MH, To KF, Chen Y, Yang W, Li MS, Shin VY, Tong JH (2015). Epigenetic silencing of miR-490-3p reactivates the chromatin remodeler SMARCD1 to promote Helicobacter pylori-induced gastric carcinogenesis. Cancer Res.

[84] Zawistowski JS, Nakamura K, Parker JS, Granger DA, Golitz BT, Johnson GL, Zawistowski JS, Nakamura K, Granger DA, Golitz BT (2013). miR-9-3p targets integrin beta 1 to sensitize claudin-low breast cancer cells to MEK inhibition. Mol Cell Biol.

[85] Higashi T, Hayashi H, Takeyama H, Kaida T, Arima K, Taki K, Okabe H, Nitta H, Hashimoto D, Chikamoto A (2015). Abstract 3125: miR-9-3p plays a tumor-suppressor role by targeting TAZ (WWTR1) in hepatocellular carcinoma cells. Br J Cancer.

[86] Zawistowski JS, Nakamura K, Parker JS, Granger DA, Golitz BT, Johnson GL (2013). MicroRNA 9-3p targets β1 integrin to sensitize claudin-Low breast cancer cells to MEK inhibition. Mol Biol.

[87] Nagel R, Sage CL, Diosdado B, Waal MVD, Vrielink JAFO, Bolijn A, Meijer GA, Agami R (2008). Regulation of the adenomatous polyposis coli gene by the miR-135 family in colorectal cancer. Cancer Res.

[88] Navarro A, Diaz T, Martinez A, Gaya A, Pons A, Gel B, Codony C, Ferrer G, Martinez C, Montserrat E (2009). Regulation of JAK2 by miR-135a: prognostic impact in classic Hodgkin lymphoma. Blood.

[89] Holleman A, Chung I, Olsen RR, Kwak B, Mizokami A, Saijo N, Parissenti A, Duan Z, Voest EE, Zetter BR (2011). miR-135a contributes to paclitaxel resistance in tumor cells both in vitro and in vivo. Oncogene.

[90] Wu S, Lin Y, Xu D, Chen J, Shu M, Zhou Y, Zhu W, Su X, Qiu P, Yan G (2012). MiR-135a functions as a selective killer of malignant glioma. Oncogene.

[91] Wang Y, Huang JW, Li M, Cavenee WK, Mitchell PS, Zhou X, Tewari M, Furnari FB, Taniguchi T (2011). MicroRNA-138 modulates DNA damage response by repressing histone H2AX expression. Mol Cancer Res.

[92] Sun Y, Chen D, Cao L, Zhang R, Zhou J, Chen H, Li Y, Li M, Cao J, Wang Z (2013). MiR-490-3p modulates the proliferation of vascular smooth muscle cells induced by ox-LDL through targeting PAPP-A. Cardiovasc Res.

[93] Honardoost M, Soleimani M, Arefian E, Sarookhani MR (2014). Expression Change of miR-214 and miR-135 during Muscle Differentiation. Cell J.

[94] He L, He X, Lim LP, de Stanchina E, Xuan Z, Liang Y, Xue W, Zender L, Magnus J, Ridzon D (2007). A microRNA component of the p53 tumour suppressor network. Nature.

[95] He X, He L, Hannon GJ (2007). The guardian’s little helper: microRNAs in the p53 tumor suppressor network. Cancer Res.

[96] Bommer GT, Gerin I, Feng Y, Kaczorowski AJ, Kuick R, Love RE, Zhai Y, Giordano TJ, Qin ZS, Moore BB (2007). p53-mediated activation of miRNA34 candidate tumor-suppressor genes. Curr Biol.

[97] Hermeking H (2009). The miR-34 family in cancer and apoptosis. Cell Death Diff.

[98] Corney DC, Hwang CI, Matoso A, Vogt M, Fleskennikitin A, Godwin AK, Kamat AA, Sood AK, Ellenson LH, Hermeking H (2010). Frequent downregulation of miR-34 family in human ovarian cancers. Clin Cancer Res.

[99] Ji Q, Hao X, Meng Y, Zhang M, Desano J, Fan D, Xu L (2008). Restoration of tumor suppressor miR-34 inhibits human p53-mutant gastric cancer tumorspheres. BMC Cancer.

[100] Okada N, Lin CP, Ribeiro MC, Biton A, Lai G, He X, Bu P, Vogel H, Jablons DM, Keller AC (2014). A positive feedback between p53 and miR-34 miRNAs mediates tumor suppression. Genes Dev.

[101] Liu N, Landreh M, Cao K, Abe M, Hendriks GJ, Kennerdell J, Zhu Y, Wang LS, Bonini NM (2012). The microRNA miR-34 modulates aging and neurodegeneration in Drosophila. Nature.

[102] Taha C, Klip A (1999). The insulin signaling pathway. J Membr Biol.

[103] Choi JH, Choi KC, Auersperg N, Leung PC (2005). Gonadotropins upregulate the epidermal growth factor receptor through activation of mitogen-activated protein kinases and phosphatidyl-inositol-3-kinase in human ovarian surface epithelial cells. Endocr Relat Cancer.

