# Supplementary material for: High-throughput sequencing of pituitary and hypothalamic microRNA transcriptome associated with high rate of egg production
Source: BMC Genomics. 2017 Mar 23;18:255. doi: 10.1186/s12864-017-3644-3 (PMC5364632; doi:10.1186/s12864-017-3644-3)
Supplement: Supplementary file 1 — The egg production distribution of 800 Luhua birds at 300 days of age. Figure S2. The egg production distribution of 400 Jiuyuan black fowls at 300 days of age. Figure S3. Pie charts of small RNA percentages. Figure S4. Length distribution of small RNA in pituitary and hypothalamic libraries of LP and HP chickens. Figure S5. Correlation analysis of miRNA expression across samples of LP and HP chickens. Figure S6. Gene ontology statistics. The GO classification map includes the top 30 significantly enriched GO terms, which can be used to highlight significant differences in gene function. (PDF 1072 kb) [file 12864_2017_3644_MOESM1_ESM.pdf]

Additional file 2: Figures S1-S6

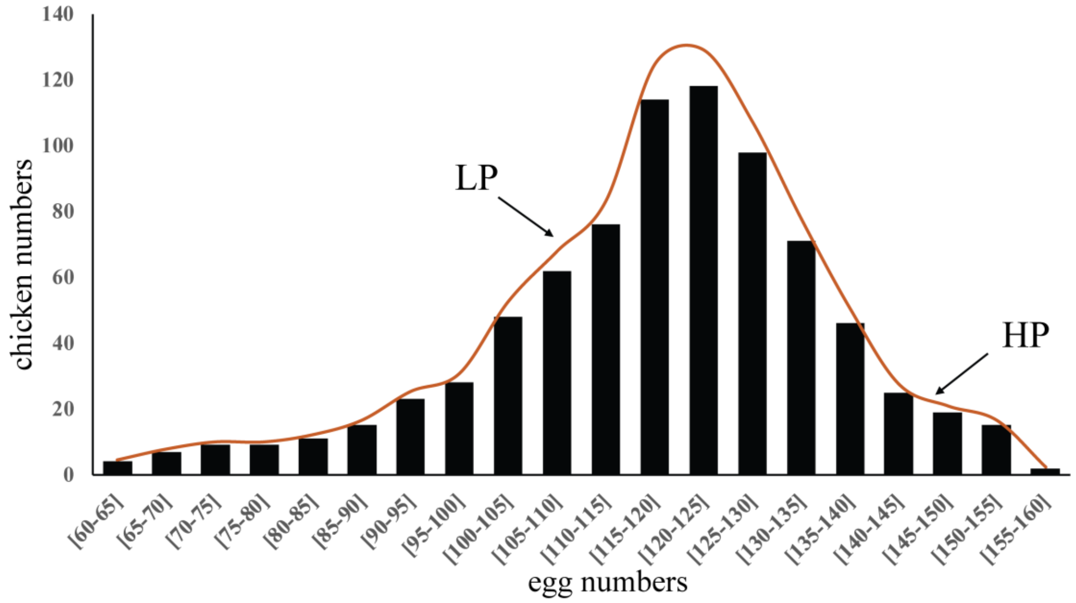

Figure S1. The egg production distribution of 800 Luhua birds at 300 days of age.

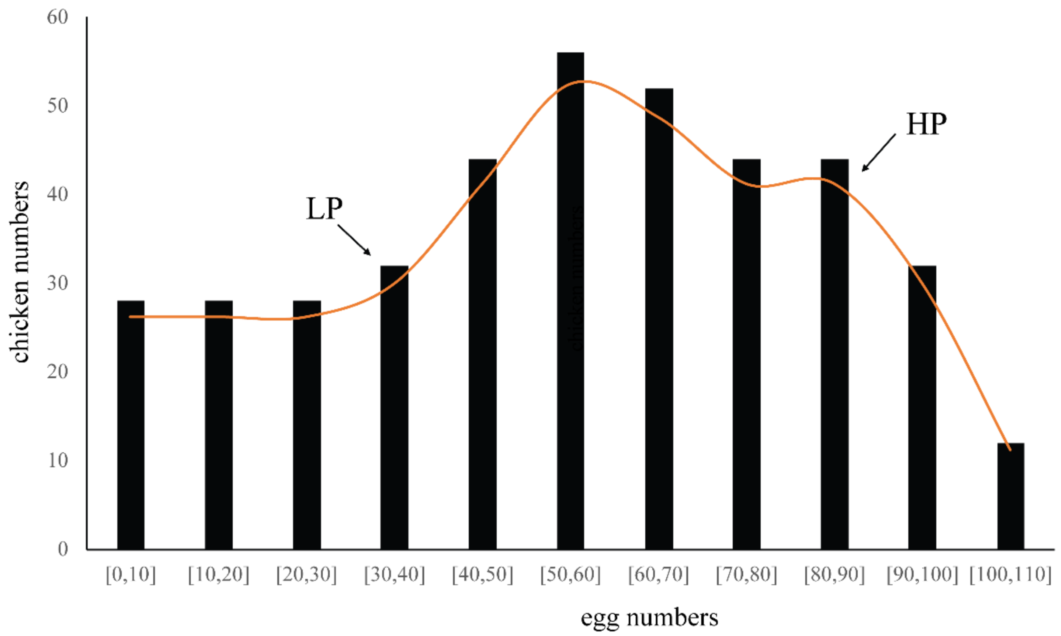

Figure S2. The egg production distribution of 400 Jiuyuan black fowls at 300 days of age.

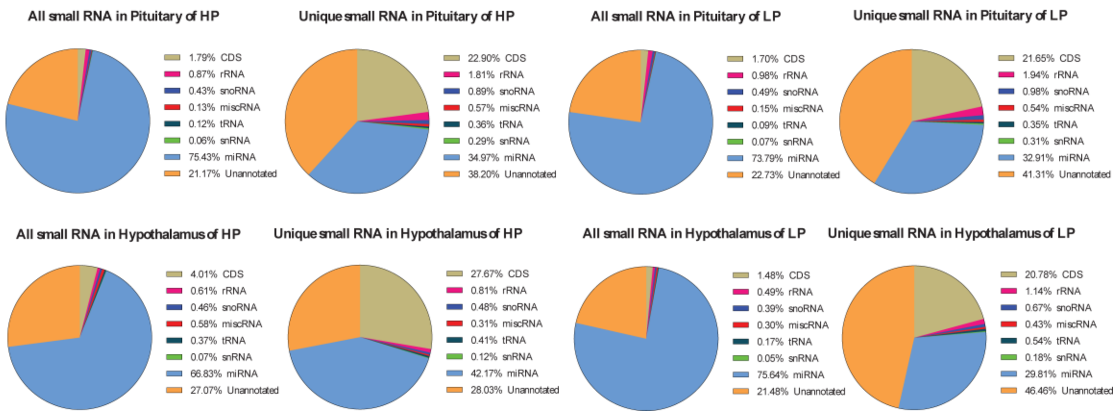

Figure S3. Pie charts of small RNA percentages.

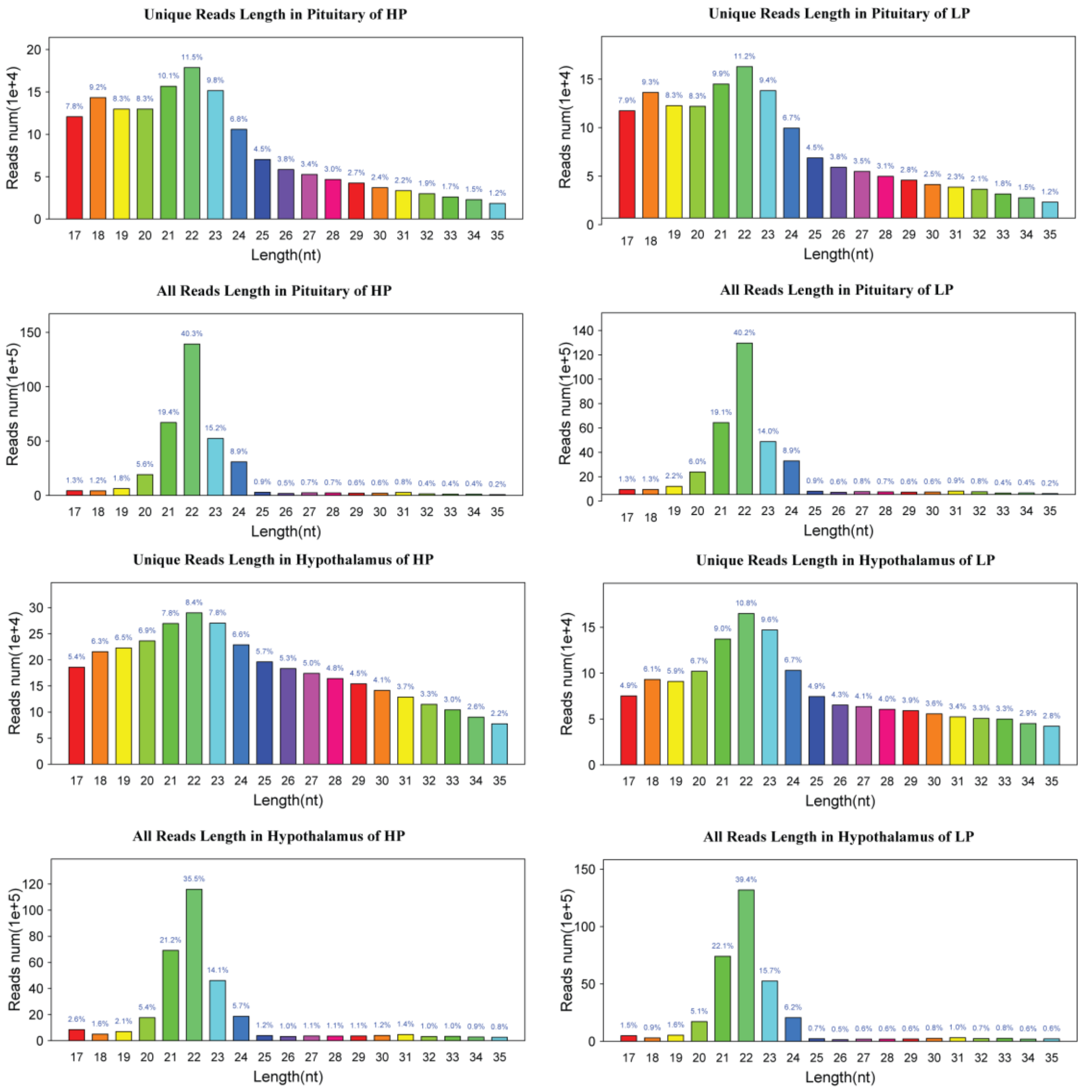

Figure S4. Length distribution of small RNA in pituitary and hypothalamic libraries of LP and HP chickens.

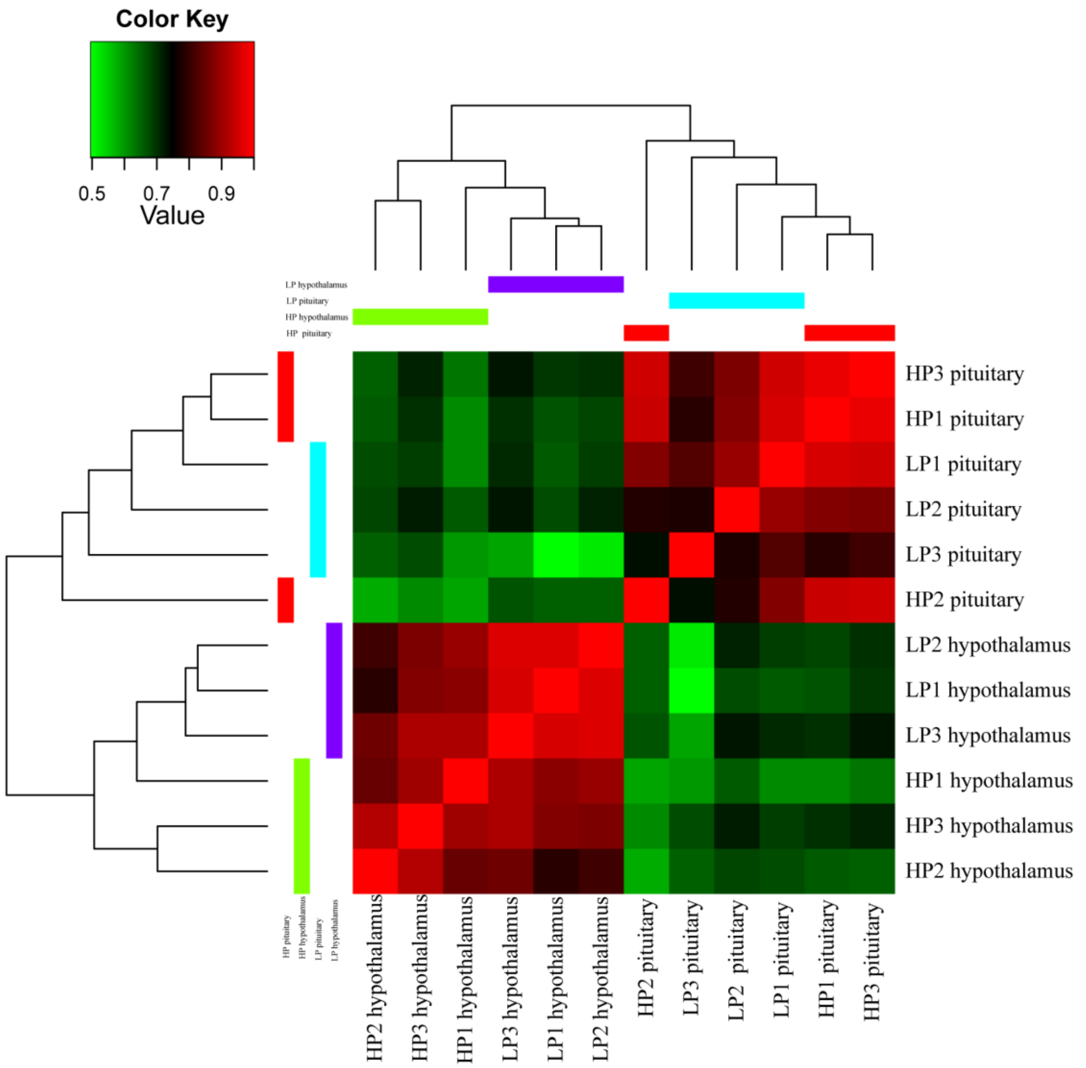

Figure S5. Correlation analysis of miRNA expression across samples of LP and HP chickens.

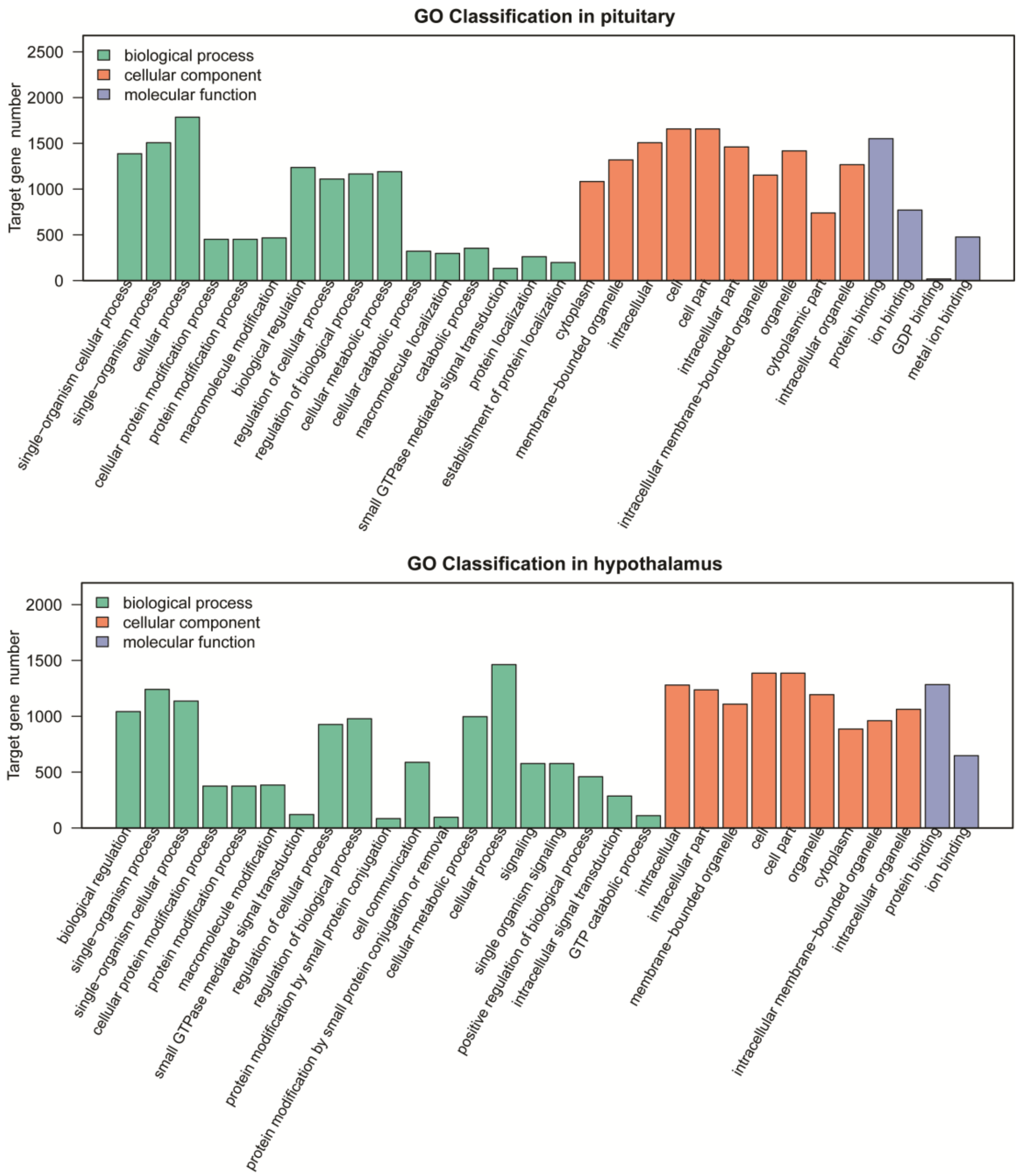

Figure S6. Gene ontology statistics.

The GO classification map includes the top 30 significantly enriched GO terms, which can be used to highlight significant differences in gene function.
